# Supplementary material for: SDF-1α-Releasing Microspheres Effectively Extend Stem Cell Homing after Myocardial Infarction
Source: Biomedicines. 2023 Jan 25;11(2):343. doi: 10.3390/biomedicines11020343 (PMC9953248; doi:10.3390/biomedicines11020343)
Supplement: Supplementary file 1 [file biomedicines-11-00343-s001.zip › biomedicines-2175559-supplementary.pdf]

Table S1. Primers used in qRT-PCR analysis.

Gene	Forward primer (5'-3')	Reverse primer (5'-3')
**SDF1α**	ACCAGTCAGCCTGAGCTACC	CACTTTAATTTCGGGTCAATGC
**CXCR-4**	TCAGTCAGGGGGATGACAGG	TGGCCCTTGGAGTGTGACAGC
**VEGFA**	AAGGAGAGCAGAAGTCCCATGA	CACAGGACGGCTTGAAGATGT
**SCF**	CTGCGGGAATCCTGTGACTG	CCAGAAGAGTAGTCAAGCTGAG
**VCAM**	TCTCTCAGGAAATGCCACCC	CACAGCCAATAGCAGCACAC
**ICAM**	GGCACCCAGCAGAAGTTGTT	CCTCAGTCACCTCTACCAAG
**MMP-9**	AGAAGCAGTCTCTACGGCCG	TGATGGTCCCACTTGAGGCC
**GAPDH**	GCACCGTCAAGGCTGAGAAC	ATGGTGGTGAAGACGCCAGT
